# Research gaps and emerging priorities in sexual and reproductive health in Africa and the eastern Mediterranean regions

**DOI:** 10.1186/s12978-018-0484-9

**Published:** 2018-03-05

**Authors:** Moazzam Ali, Madeline Farron, Leopold Ouedraogo, Ramez Khairi Mahaini, Kelsey Miller, Rita Kabra

**Affiliations:** 1 Department of Reproductive Health and Research, World Health Organization, Geneva, Switzerland; 2 Reproductive and Women’s Health, Africa Region, World Health Organization, Brazzaville, Democratic Republic of the Congo; 3 Women’s Reproductive Health, Eastern Mediterranean Region, World Health Organization, Cairo, Egypt

**Keywords:** Research priority, Maternal health, Adolescent, Contraception, Eastern Mediterranean, Africa

## Abstract

**Background:**

In-country research capacity is key to creating improvements in local implementation of health programs and can help prioritize health issues in a landscape of limited funding. Research prioritization has shown to be particularly useful to help answer strategic and programmatic issues in health care, including sexual and reproductive health (SRH). The purpose of this paper is to present the results of a priority setting exercise that brought together researchers and program managers from the WHO Africa and Eastern Mediterranean regions to identify key SRH issues.

**Methods:**

In June 2015, researchers and program managers from the WHO Africa and Eastern Mediterranean regions met for a three-day meeting to discuss strategies to strengthen research capacity in the regions. A prioritization exercise was carried out to identify key priority areas for research in SRH. The process included five criteria: answerability, effectiveness, deliverability and acceptability, potential impact of the intervention/program to improve reproductive, maternal and newborn health substantially, and equity.

**Results:**

The six main priorities identified include: creation and investment in multipurpose prevention technologies, addressing adolescent violence and early pregnancy (especially in the context of early marriage), improved maternal and newborn emergency care, increased evaluation and improvement of adolescent health interventions including contraception, further focus on family planning uptake and barriers, and improving care for mothers and children during childbirth.

**Conclusion:**

The setting of priorities is the first step in a dynamic process to identify where research funding should be focused to maximize health benefits. The key elements identified in this exercise provides guidance for decision makers to focus action on identified research priorities and goals. Prioritization and identifying/acting on research gaps can have great impact across multiple sectors in the regions for improved reproductive, maternal and children health.

## Background

Research capacity building in health fields is important specially in low and middle-income countries (LMICs). According to the World Health Report 2013 it can strengthen health systems and can help move countries towards universal health coverage [1]. Despite the large body of scientific research, protocols and strategic models to address health problems, there is an increased need for better implementation methods in order to make an impact on health outcomes. Through increased research capacity building, implementation of existing strategies and scaling up interventions may find more success as local researchers bring local knowledge and much needed perspective to these endeavors. Research capacity can also improve health system development, inform more effective policy and lead to better governance in individual countries [2].

Research capacity building on the African continent has particularly great potential as Africa has the greatest burden of disease and the lowest-density of healthcare professionals in the world [3]. Shortages of researchers, faculty members, infrastructure, and a dearth of career opportunities for upcoming researchers in Africa to build up a critical mass of scientists to prioritize and carry out policy relevant research exists [3]. Moreover, multiple factors act as barriers and issues to the health system, hindering the ability of effective health care to be delivered in these contexts. One of the largest complications is that numerous, diverse issues have been identified as health care priorities in the region, which has led to a competition for resources [4]. This lack of a coherent message is exacerbated by a lack of political will, infrastructural inadequacies, and logistical weaknesses [4] thereby paralyzing action.

Research priority setting is acknowledged to be a key function of national health research systems and is perceived to be an important process in terms of ensuring the alignment of research funding with national evidence needs [5]. It is usually done at different hierarchical levels of the health research system (national, institutional, departmental, or at a program level). Ideally health policy and systems research priorities would emerge through priority setting processes. However priority setting for health research is often not performed well or not performed at all [6]. Priorities need to be reviewed and updated periodically. Through prioritization, SRH could be given more attention, research, and action providing a great benefit to individuals in Africa.

Research prioritization is one of the key nodal points in the research cycle, which includes research planning, research priority setting, strategies and implementation of research priorities, research utilization, research monitoring and evaluation (part of the research information system) and overall research management. The final aim of research prioritization is how “balanced research can support and complement the health system to achieve the national goals for health.” With research prioritization, a forward-looking research system can be firmly established [7].

Research can play a critical role in the response to global health challenges. But when resources are limited, guidelines are needed to assist decisions on defining the priorities for health research investments [8]. Setting priorities for health research is essential to maximize the impact of investments, which is especially relevant in resource-poor environments [9–11].

In June 2015, the WHO/HRP’s Regional Committee meeting for the African and Eastern Mediterranean regions on research capacity strengthening in Sexual and Reproductive Health and Rights (SRH) met in Nairobi, Kenya. The three-day meeting was attended by 38 participants, including 17 women, who represented the research partners of the HRP, the collaborating centers of the WHO, the country offices of WHO, the regional offices of the WHO, the long-term institutional development grantees, and the staff from headquarters in Geneva. The main purpose of this meeting was to discuss the issues related to research capacity strengthening and the future research priorities in sexual and reproductive health and rights in the area and for the WHO.

The meeting was dedicated to discussing the challenges and the lessons learned regarding research capacity strengthening in the regions. This discussion included the emerging research priorities in the two regions. The research prioritization session was mainly dedicated to aid countries in prioritizing their research goals and to identify the main SRH research priorities for the African and Eastern Mediterranean Regions.

The discussion focused on regional experiences on research implementation plans and strategies for strengthening research capacity in SRH, identification of potential barriers and challenges inherent in these proposed plans. The challenges included lack of adequate funding, inadequate capacity to support research, regional brain drain in LMICs, poor communication within the WHO, understaffing, inadequate involvement of policy makers, and poor dissemination and use of research results.

The main objective of this paper is to present the findings of the exercise in identifying an actionable, prioritized research agenda on sexual and reproductive health in the WHO African and Eastern Mediterranean Regions.

## Methods

Priorities for research on SRH were identified in three main stages in our exercise. In the first stage, the group of researchers, program managers, and other stakeholders from the African and Eastern Mediterranean regions were provided with an overview on various prioritization techniques. The framework of prioritization was presented which included five criteria: answerability (likelihood that research question can be answered ethically), effectiveness (likelihood that the new knowledge would lead to an effective intervention or program), deliverability and acceptability (likelihood that the intervention or program would be deliverable and affordable), potential impact (likelihood that the intervention or program could improve maternal and newborn health substantially), and equity (likelihood that the intervention or program will reach the most vulnerable groups).

Following the discussion of these criteria for prioritization, participants were divided into groups for the prioritization activity. In the second stage, a broad list of sample topics within sexual and reproductive health and maternal and child health were offered as jumping off point. Each group using the prioritization criteria came up with five priorities after deliberation and discussion that were then presented to the entire group. In the third and final stage, the three groups’ priorities were discussed in the large group and a consensus was reached on the six main priorities presented below by attendees.

## Results

The aim of this exercise was to create a comprehensive set of broad goals with actionable priorities to combat the problems identified in SRH as recognized by the participating members. The goals were identified to be broad, focusing more on overarching trends of need in the region and in the field of sexual and reproductive health in general. The participants were able to identify main goals that addressed these broad trends and needs in the region.

Three main high-level goals were identified for both of the regions: quality of care, contraception, and adolescent health.

The first two goals relate to sexual education and contraception. The first goal emphasizes early adolescent sexuality education in out of school, and the delay of sexual activity for all adolescents. The second goal is the development of contraception services, including post-partum and post-abortion services. This second goal also aims to address barriers to contraceptive methods, including long-acting reversible contraceptives (LARCs) and emergency contraception.

The third goal is the development of quality of care in three areas: childbirth, general sexual and reproductive services, and disrespect or abuse in childbirth. This also includes improving Emergency Obstetric Care, covering multiple topics, including blood practices, organization of services, assisted vacuum delivery, and unsafe abortion practices.

### Priorities and actions from the goals identified

In addition to developing the above broad goals, the team also created a list of priorities for future SRH research. The list of priorities was extensive and comprehensive for sexual and reproductive health research in the region. While all the priorities are important and will play a major role in the future of sexual and reproductive health research in the region, the top six research priorities are given increased attention based on their effectiveness to improve sexual and reproductive health in the regions.

The below mentioned six priority areas were highlighted and selected by the meeting participants as the most pressing and prioritized aspects of sexual and reproductive health to be addressed in the near future. The priorities are written in no particular order and carry equal weight.

**The first priority area is the creation and investment in multipurpose prevention technologies.** This is especially relevant in the context of condoms and their unique place in sexual and reproductive health. Condoms are one of the major prevention techniques for two pressing issues in SRH: family planning and HIV/AIDS prevention. Because condoms are used in both contexts they are the only examples of a multipurpose sexual health technology. While this is a new field, there is potential in expanding research in this area to create more technologies that can address multiple issues and move toward a more comprehensive sexual and reproductive health product market.

**The second priority area is addressing adolescent violence and preventing early pregnancy using contraception, particularly in the context of early marriage**. Adolescent violence includes sexual violence, physical violence, and psychological violence. Early marriage greatly affects SRH as girls have an earlier sexual debut, give birth to more children, have higher mortality and morbidity rates (with pregnancy being the leading cause of death for women 15-19), have higher infant and child mortality rates, have an increased risk of experiencing partner violence, and affects educational opportunities for the girl [12]. The participants wanted to prioritize girls aged 10-14 years old and increase access to services to delay marriage, first births, and violence for women in or out of relationships.

**The third priority is to increase the quality of care and safety associated with maternal and newborn emergency care in the region, and more specifically a focus on blood products and the organization of maternal services**. Blood services and products are a priority for multiple health outcomes. Practices for blood transmission and safety in pregnancy are a concern, particularly when it comes to minimizing transmission of blood-borne illness and viruses (particularly HIV and hepatitis) from mother to child. The standards and practices surrounding the quality and safety of blood in hospitals and health care facilities need to be examined and improved including: examination and improvement of the systems surrounding acquisition of blood, storage of blood, transport of blood and proper documentation and data analysis of all blood product-related health care practices.

**The fourth priority is to evaluate and improve adolescent health interventions in and out of schools in the region.** This is to include the promotion and utilization of comprehensive sexual education and the human rights based approach for students and youth in general. This will include a curriculum that uses a comprehensive sex education program, and will emphasize information on menstruation, menstrual hygiene, puberty and access to contraception as key aspects to delivering the best health care to adolescents. Menstrual care and hygiene is specifically important for adolescent girls leading to overall increased health and dignity, as promoted by the current ELRHA toolkit [11].

**The fifth priority is to focus on family planning uptake, methods used, and engagement.** It was recognized that it was essential to ensure access to and availability of effective contraceptive methods to all. Participants advocated for increased usage and also identified many of the barriers to FP with possible solutions. Mixed method use and additional contraception options are needed (including lactational amenorrhea and IUDs). Access to emergency contraception was acknowledged as a priority as well as post-partum and post-abortion family planning counseling. Finally, participants felt male involvement should be emphasized in the regions and discussed ways to engage men.

**The sixth priority identified focuses improvement of the services, practices and quality of care for both mother and newborn during child birth**. This includes promotion of companionship in birthing services. This companionship support will encompass both the presence of fully trained community health workers in the pre-natal and birthing processes, as well as encourage the full support and participation of fathers in both the prenatal stage and at the time of delivery. The trained health workers will be able to support pregnant women, provide respectful care as well as properly refer women to hospital care, as well as provide prenatal support and counselling. Organization and standardization of childbirth care facilities were also prioritized in order to provide improved care, service, and safety to clients. This relates to other priorities identified such as elimination of obstetric fistulas and management of postpartum hemorrhages.

**Other priorities identified** included increased attention to cervical cancer including treatment and prevention with the HPV vaccination, need of more reliable data and studies regarding STI prevalence in the populations for the regions.

Additional priorities included general commitments to increasing quality of care, access, and increased impact evaluation to identify and implement best practices for SRH. Participants identified task-shifting as a possible solution. Participants prioritized the need to improve the access to reproductive health services for women with disabilities in the region. Finally, there was a priority established to address and combat violence against women in all forms and in all populations.

## Discussion

In the past two decades since the ICPD’s Cairo consensus, research has helped to define what works and at what cost to improve sexual and reproductive health. However, the remaining gaps in our knowledge and understanding are substantial, and impede greater progress and success. Conducting prioritization exercises will assist the regions and countries to understand (i) the full spectrum of research investment options, (ii) the potential risks and benefits that can result from investments in different research options, and (iii) the likelihood of achieving reductions in the persisting burden of maternal and child health morbidity and mortality. Increasingly, there is a need for national governments, public-private partnerships, private sector and other funding agencies to set priorities in health research investments in a fair and transparent way.

There are many approaches to health research prioritization. The identification of common themes for good practice fulfils the need for guidance on this varied and intricate process. The opportunity allowed participants to thoroughly think about the role of SRH in their own health systems and how they would prioritize and improve research capacity building and move forward with possible evidence-based policy solutions or interventions addressing the identified priorities. By identifying goals and priorities, governments may realize the importance of developing research capacity in their own countries in order to produce more relevant solutions and improved implementation in country. The prioritization exercise may also be applied to other health issues in countries since there are competing health issues and limited funding.

Governments should invest in prioritizing research in their own countries and follow through with their goals by increasing focus on sexual and reproductive health issues in their own countries, which can only be developed and implemented with strong research capacity. There should also be additional in-country research on the six priorities mentioned. With this research on these prioritized areas, additional context-specific implementation strategies can be developed and a new, expanded research culture may flourish in the regions. The setting of research priorities is the first step in a dynamic process to identify where research funding should be focused to maximize health benefits. It is important to realize that these results represent a regional discussion of the issues in sexual and reproductive health. They should be interpreted carefully when applied at the country levels because of the differences in needs and context of individual countries.

## Conclusion

In conclusion, respective governments must seriously invest in research capacity in order to create a critical mass of researchers in country who can do prioritization and help create impactful and useful policy for the local context. Prioritization is key to taking action in the face of limited funding. Investing in this cadre of researchers will lead to more successful implementation compared with foreign researchers providing implementation advice, as local researchers know their communities and countries best. This is particularly important in the realm of sexual and reproductive health as it has been underserved. With prioritization and research capacity, governments make headway on improving quality, care, and health of their citizens.

The prioritization exercise helped identify concrete issues for action and implementation. Hopefully, more attention and funding can be shifted towards this useful and often neglected element of research development. Further research is needed to determine how best to evaluate success of priority setting at country and regional levels.

## Abbreviations

HRP: Human reproduction program
ICPD: International Conference on Population and Development
IUDs: Intra uterine device
LARC: Long-acting reversible contraceptives
LMICs: Low and middle-income countries
SRH: Sexual and reproductive health
STI: Sexually transmitted infections
WHO: World Health Organization
